# Proposal for defining the severity of environmental hypoxia

**DOI:** 10.3389/fphys.2026.1985822

**Published:** 2026-09-25

**Authors:** Martin Burtscher, Ricardo Amaru, Johannes Burtscher, Katharina Hüfner, Max Gassmann

**Affiliations:** 1 Institute of Sport Science, University of Innsbruck, Innsbruck, Austria; 2 Cell Biology Unit, National Academy of Sciences, Universidad Mayor de San Andres, La Paz, Bolivia; 3 Department of Psychiatry, Psychotherapy, Psychosomatics and Medical Psychology, University Hospital for Psychiatry II, Medical University of Innsbruck, Innsbruck, Austria; 4 Institute of Veterinary Physiology, Vetsuisse Faculty, University of Zurich, Zurich, Switzerland; 5 The Zurich Center for Integrative Human Physiology (ZIHP), University of Zurich, Zurich, Switzerland; 6 Universidad Peruana Cayetano Heredia, Lima, Peru

**Keywords:** acclimatization, altitude, definition, hypoxia severity, normobaric

## Abstract

Every day, millions of people are exposed to environmental hypoxia, primarily due to living at or travelling to high altitudes for reasons such as work, tourism, business, or sports, e.g., mountaineering. Furthermore, hypoxic exposure at low altitudes (e.g., in hypoxia rooms or tents) is increasingly being used to pre-acclimatize individuals to high altitudes for activities such as athletic training, trekking and high-altitude climbing, as well as for preventive and therapeutic purposes. Especially for these applications a clear definition of environmental hypoxia severity is lacking, impeding risk assessment. Our proposed classification does not address the acclimatization process itself, but rather the degree of hypoxia that healthy individuals can tolerate acutely (for a few hours) without prior acclimatization and chronically (up to years) after acclimatization.

Based on real-world levels of hypoxia observed in high-altitude residents and mountaineers, as well as in individuals exposed to simulated hypoxia at low altitude, we propose the following definitions: Altitudes of up to 1,000 m, corresponding to fractional inspired oxygen (FiO_2_) levels above 18.5%, should be considered ‘normoxia’. Altitudes ranging from 1,000 m to 3,500 m or FiO_2_ levels between 18.5% and 13.5% can be classified as mild hypoxia. Altitudes between 3,500 m and 5,500 m or FiO_2_ levels between 13.5% and 10.2%, represent moderate hypoxia. While people usually can acclimatize to mild and moderate hypoxia, the severe hypoxia experienced at altitudes above 5,500 m or FiO_2_ levels below 10.2% poses considerable health risks to humans, where acclimatization in the long term is no longer possible.

## 1 Introduction

Millions of people are exposed to environmental hypoxia (low levels of oxygen, O_2_) every day, most of them because they live at or travel to high altitudes for work, sightseeing, business, climbing mountains, skiing and many other reasons (Burtscher et al, 2023). Moreover, hypoxic exposure at low altitude is increasingly applied to prepare individuals for high-altitude stays, either for athletic training, trekking or climbing, for preventive and therapeutic purposes or for research (Boulares et al, 2025; Burtscher et al, 2026; Feng et al, 2024; Rybnikova et al, 2022).

However, no broadly accepted classification of environmental hypoxia severity and its associated physiological conditions, or its potential consequences currently exists. The hypoxia levels that we propose here refer to the physiological conditions encountered by healthy adults during short-term hypoxic exposure, prior to the development of altitude illness, and following adequate acclimatization to prolonged hypoxic exposure. The acclimatization process itself, including the associated risk of developing altitude illness, is not considered in this classification because it varies considerably between individuals and represents a transitional, time-dependent process rather than a stable level of hypoxia tolerance. For comprehensive guidance on individualized acclimatization strategies, particularly for high-altitude mountaineers, we refer readers to previous work (Burtscher et al, 2026; Gatterer et al, 2024; Luks et al, 2024).

Since blood O_2_ levels in healthy adults commonly remain normal up to an altitude of at least 1,000 m, and complete acclimatization is possible up to an altitude of around 5,000 - 5,500 m (Champigneulle et al, 2024; Luks et al, 2024), we propose subdividing this range into mild and moderate hypoxia. Accordingly, conditions below 1,000 m are considered normoxic, whereas altitudes above 5,500 m are associated with severe hypoxia, which is not compatible with permanent residence and is associated with a substantially increased risk of adverse health events. To support this proposal and justify the ranges defined for mild and moderate hypoxia, we draw on physiological data from acute, subacute, and chronic exposure to altitude, together with evidence on the tolerability of different levels of hypoxia under defined exposure conditions.

## 2 Current classifications of altitude zones and hypoxia levels

While there is a reasonable consensus regarding the classification of altitude zones, this does not apply to hypoxia levels, particularly when applied at simulated altitude (i.e., normobaric hypoxia). At terrestrial altitudes the level of hypoxia can be defined based on the actual barometric pressure (P_B_) and the related O_2_ partial pressure (PO_2_), both declining with increasing altitude (hypobaric hypoxia). The percentage of O_2_ in the air remains almost constant at around 21% as altitude increases. For normobaric hypoxia (i.e., instead of lowered P_B_, the concentration of inspired PO_2_ is reduced), the corresponding hypoxia level is frequently expressed as the fraction of inspiratory O_2_ (FiO_2_). Common classifications of altitude zones define “low altitude” up to 1,000 or 1,500 m, “moderate altitude” from 1,000 or 1,500 m to 2,500 m, “high altitude” from 2,500 to 3,500 m, “very high altitude” from 3,500 to 5,500 m, and “extreme altitude” above 5,500 m (Gatterer et al, 2024; Hackett and Roach, 2001; Mallet et al, 2025). Moderate altitudes have also been defined to range between 2,000 and 3,000 m when considering athletes, i.e. soccer players (Bärtsch et al, 2008). All of these altitude classifications have their merit and are adequate for their respective applications. However, ‘hypoxia’ (normobaric or hypobaric) has been categorized inconsistently across studies and applications. We therefore focus primarily on environmental or inspiratory hypoxia levels encountered at terrestrial or simulated altitudes.

A recent paper describes minimal hypoxia as an FiO_2_ of 0.209 to 0.17 (simulated altitude of 0 to 1,700 m), moderate hypoxia as 0.17 to 0.10 (simulated altitude of 1,700 to 5,600 m), and extreme hypoxia as FiO_2_ values below 0.10 (above 5,600 m) (Rogers and Mootha, 2025). Another group classified FiO_2_ values between 0.16 and 0.09 as moderate hypoxia (Rybnikova et al, 2022), and yet another recently published paper even suggests that an FiO_2_ of 0.10 to 0.08 is ‘mild’ hypoxia (Wszędybył-Winklewska et al, 2026). Sometimes the severity of hypoxia is not defined at all, but instead the normal range of peripheral oxygen saturation (SpO_2_) at different altitudes, i.e. > 96% at sea level, 93-97% at 1,500 m, 90-95% at 2,500 m, 82-88% at 3,500 m, 75-85% at 4,500 m, and 70-80% at 5,500 m is referred to (Cai et al, 2026). The latter may provide a useful foundation for the classification of environmental hypoxia, which could be reinforced by allocating blood gas values and hypoxia severity levels to different altitudes or FiO_2_ values. For example, an SpO_2_ of approximately 90% at 3,500 m corresponds to an arterial PO_2_ (PaO_2_) of approximately 60 mmHg (based on the standard oxyhemoglobin dissociation curve), a clinically relevant threshold generally considered to mark the onset of hypoxemia.

## 3 Physiological considerations

P_B_ and PO_2_ decrease with increasing terrestrial altitude. Due to the sigmoid-shaped hemoglobin-O_2_ dissociation curve, O_2_ saturation of the arterial blood (SaO_2_) or the estimated peripheral O_2_ saturation (SpO_2_) decreases only moderately up to an altitude of around 2,000 to 2,500 m or a PaO_2_ of approximately 60–70 mmHg, after which the slope of the decrease becomes considerably steeper (Burtscher et al, 2025a). This may explain why adverse health effects of hypoxia are rare up to these altitudes and why hypoxic exposure at this level can even provide a stimulus for beneficial physiological adaptation (Burtscher et al, 2025b). To estimate values of the PaO_2_ and SpO_2_ for different altitudes and compare them with available data taken at different altitudes, it is helpful to examine the O_2_ cascade from ambient air to the arterial blood, ultimately culminating in cellular O_2_ consumption, primarily by mitochondrial respiration (Samaja and Ottolenghi, 2023). Based on the alveolar gas equation, the alveolar partial pressure of O_2_ (PAO_2_) is defined as:

Equation 1: $PAO_{2}=\;\left[FiO_{2}x\;\left(P_{B}–\;47\right)\right]\;–\;PaCO_{2}/R)$where P_B_ indicates the atmospheric pressure (760 mmHg at sea level), and “47” the partial pressure of water at 37 °C, both in mmHg; R represents the Respiratory Quotient (at the cellular level) but in practice the Respiratory Exchange Ratio (as measured at the lungs) is used. PaCO_2_ is the arterial partial pressure of carbon dioxide.

Depending on the efficiency of pulmonary gas exchange, there is a certain difference in O_2_ pressure between the alveoli (PAO_2_) and arterial blood (PaO_2_). This alveolar-arterial O_2_ difference in a healthy, young and resting person at sea level amounts to about 5–10 mmHg, and this value may be even lower at higher altitude, i.e., between 0–5 mmHg (Calbet and Lundby, 2009), but increases with age. Thus, assuming sea-level PaCO_2_ is maintained, PaO_2_ would decline to approximately 95, 40, and 17 mmHg at sea level, 3,500 m, and 5,500 m, respectively. However, in response to reduced oxygen availability, hypoxia-induced hyperventilation lowers PaCO_2_, thereby partially preserving arterial oxygenation. For example, if PaCO_2_ decreases to 25 mmHg, the corresponding calculated PaO_2_ values would increase to approximately 60 and 37 mmHg at 3,500 and 5,500 m, respectively. Such hypocapnia may occur during acute exposure to hypoxia (minutes to hours) but is increasingly constrained by the resulting respiratory alkalosis. With prolonged exposure, renal bicarbonate excretion progressively compensates for the respiratory alkalosis, thereby reducing its inhibitory effect on ventilation and allowing sustained increases in minute ventilation as part of ventilatory acclimatization.

Because a PaO_2_ below 60 mmHg is generally considered indicative of hypoxemia, an altitude of approximately 3,500 m (FiO_2_ = 0.135) may serve as a physiologically meaningful threshold for categorizing hypoxia. This applies to both short- and long-term exposure with appropriate ventilatory compensation, but not to the acclimatization phase.

Notably, the simulated altitude indicated by the FiO_2_ values is only valid if FiO_2_ is applied at sea level. If certain FiO_2_ values are applied in elevated areas, the simulated P_B_ equals the P_B_ of residence x FiO_2_ applied/0.209. The simulated altitude can then be derived using air pressure–altitude tables or calculators available online. For example, an FiO_2_ of 0.135 at sea level corresponds to a simulated altitude of 3,500 m. However, the same FiO_2_ at an altitude of 1,000 m corresponds to a simulated altitude close to 4,500 m!

## 4 Real world hypoxemia levels based on blood gas analyses at various altitudes

Several examples illustrate variations in mean values of hypoxemia levels at different altitudes, both for people exposed to simulated altitude, ascending to real high altitudes and for those living there permanently. Together, they help to establish a basis for categorizing environmental hypoxia as mild, moderate, or severe. Data are summarized in **Table 1**.

**Table 1:** Summary of data on real-world hypoxemia levels.

Reference	Population	Altitude (m)	Time at altitude	PaO_2_ (mmHg)	PaCO_2_ (mmHg)
Forrer et al. (2023, systematic review; data estimated)	Healthy lowlanders	2,000	Within first 3 days	76	—
Cid-Juárez et al. (2023)	Mexico City residents	2,240	Permanent residence	67	31
Forrer et al. (2023, systematic review; data estimated)	Healthy lowlanders	2,500	Within first 3 days	70	—
Skalla et al. (2026)	Healthy lowlanders	3,100	24 h	59.5	32
Forrer et al. (2023, systematic review; data estimated)	Healthy lowlanders	3,500	Within first 3 days	58	—
Zubieta-Calleja & Zubieta-DeUrioste (2023)	La Paz residents	3,500	Permanent residence	~60	—
Tymko et al. (2022)	Lowlanders	3,800	Several days (acclimatized)	54	31
Tymko et al. (2022)	Andean highlanders	3,800	Permanent residence	51	31
Tymko et al. (2022)	Lowlanders	4,300	Several days (acclimatized)	49	28
Tymko et al. (2022)	Andean highlanders	4,300	Permanent residence	47	32
Savourey et al. (2007)	Healthy lowlanders	4,500 (hypobaric simulation)	40 min	48	—
Savourey et al. (2007)	Healthy lowlanders	4,500 (normobaric simulation)	40 min	52	—
Forrer et al. (2023, systematic review; data estimated)	Healthy lowlanders	4,500	Within first 3 days	46	—
Tymko et al. (2022)	Lowlanders	5,000	Several days (acclimatized)	44	29
Tymko et al. (2022)	Andean highlanders	5,000	Permanent residence	35	34
Champigneulle et al. (2024)	La Rinconada residents	5,000–5,300	Permanent residence	~35	—
Forrer et al. (2023, systematic review; data estimated)	Healthy lowlanders	5,500	Within first 3 days	34	—

Average values are provided for partial pressure of oxygen (PaO_2_) and carbon dioxide (PaCO_2_).

Individual PaO_2_ values vary considerably during acute exposure to terrestrial or simulated altitude, but mean values are broadly comparable with those reported after several days or longer-term exposure. Across studies in lowlanders and high-altitude residents, PaO_2_ decreases approximately linearly between 2,000 and 5,500 m, with no consistent evidence of major differences related to exposure duration or acclimatization. This may partly reflect similar ventilatory responses at the time of measurement, either before substantial respiratory alkalosis develops or after renal compensation has attenuated its inhibitory effect on ventilation. The available data therefore support 3,500 m as a practical threshold for mild hypoxia, as PaO_2_ generally remains at or near 60 mmHg, the commonly accepted threshold for hypoxemia, up to this altitude. Above approximately 5,000 - 5,500 m, PaO_2_ may fall below 35 mmHg, approaching a level considered incompatible with sustained human life. These values only apply to healthy adults of both sexes under resting or near resting conditions and are estimated averages. It is well known that oxygen demand is increased and arterial hypoxemia is exacerbated by exercise in hypoxia (Bärtsch and Swenson, 2013). Individual hypoxia responses vary greatly and especially in individuals with pre-existing conditions, such as chronic respiratory diseases. Even mild environmental hypoxia can severely impair the health of these individuals (Bloch et al, 2023).

## 5 Hypoxia tolerance with regard to altitude/hypoxia levels

Evidence for the tolerability of hypoxia corresponding to approximately 3,500 m comes from real-world observations in large populations living at such altitudes. For example, more than 750,000 people live, work, and engage in physical activity in La Paz, Bolivia, at an altitude of approximately 3,500 m. This also applies, in general, to La Rinconada, the highest city in the world, which lies almost 2,000 m higher than La Paz, at approximately 5,100–5,300 m. However, long-term residence at this extreme altitude is associated with substantial health burdens, including a considerable prevalence of chronic mountain sickness (CMS) (Savina et al, 2024). The CMS prevalence which was reported to increase from 2–3% at around 3,500 m to more than 30% at 5,500 m in young Chinese male immigrants in Tibet provides support for the proposed categorization (Pei et al, 2012).

Altitudes up to about 5,500 m are generally well tolerated by most individuals acclimatized to those altitudes (Calbet et al, 2002; West, 2006), but also by unacclimatized healthy individuals, at least for short exposures, i.e., applied intermittently or continuously for a few hours (Burtscher et al, 2014; Serebrovskaya and Xi, 2016). In a study of 77 healthy men and women, we demonstrated that even individuals susceptible to AMS tolerated a 3-hour continuous exposure to a simulated altitude of approximately 4,500 m (FiO_2_ = 0.126) at rest without developing clinically significant symptoms of altitude illness. Such short exposures are increasingly used for training, preventive or therapeutic purposes (often intermittently) (Boulares et al, 2025; 2024; Burtscher et al, 2024; Serebrovskaya, 2002; Zhang et al, 2023). It has repeatedly been demonstrated that healthy individuals can safely be exposed to hypoxic levels corresponding to FiO_2_ values of 0.10 - 0.11 under resting conditions, for both short and even longer periods (5 days) when gradually adjusted (Berra et al, 2024; Chacaroun et al, 2016).

Effects of sex and age: Sex modifies several physiological responses to hypoxia, including ventilatory control, pulmonary vascular reactivity, exercise-induced oxygen desaturation, and susceptibility to altitude illness. Women may experience greater hypoxemia during exercise, whereas their pulmonary vascular response to hypoxia is generally less pronounced (Raberin et al, 2024). Despite substantial sex differences in baseline hemoglobin concentration and iron stores, hypoxia-induced erythropoietic adaptation appears broadly similar between women and men, although limited iron availability may more frequently constrain adaptation in women (Raberin et al, 2024). Aging may likewise influence hypoxia tolerance, particularly through age-related reductions in ventilatory, cardiovascular, and gas-exchange reserve. However, in healthy, physically active individuals, aging does not appear to impair the overall cardiac or ventilatory response to hypoxia, at least up to the eighth decade of life (Lhuissier et al, 2012). Thus, these observations do not warrant sex- or age-specific categories of hypoxia severity, provided individuals are healthy, physically active, and free from relevant acute or chronic disease.

## 6 Proposed categorization and practical guidance

Based on real-world observations of arterial oxygenation and the tolerability of altitude and hypoxic exposure, we propose a four-level categorization of environmental oxygen availability: normoxia below approximately 1,000 m (FiO_2_ > 0.185), mild hypoxia from 1,000 to 3,500 m (FiO_2_: 0.185 – 0.135), moderate hypoxia from 3,500 to 5,500 m (FiO_2_: 0.135 – 0.102), and severe hypoxia above 5,500 m (FiO_2_< 0.102) (**Figure 1**). These thresholds are intended as physiologically informed reference points and not as absolute biological boundaries. The proposed categorization describes the severity of the environmental hypoxic stimulus, which is generally well tolerated during short-term exposure (up to approximately 3 hours) and, after adequate acclimatization, during prolonged exposure. Tolerability is generally greater up to approximately 3,500 m (FiO_2_: 0.135) than at 3,500 - 5,500 m (FiO_2_: 0.135 – 0.102), where the physiological strain is higher. The categorization is not necessarily valid during the acclimatization process itself, because it is highly individual and depends on the severity and duration of exposure.

**Figure 1:**
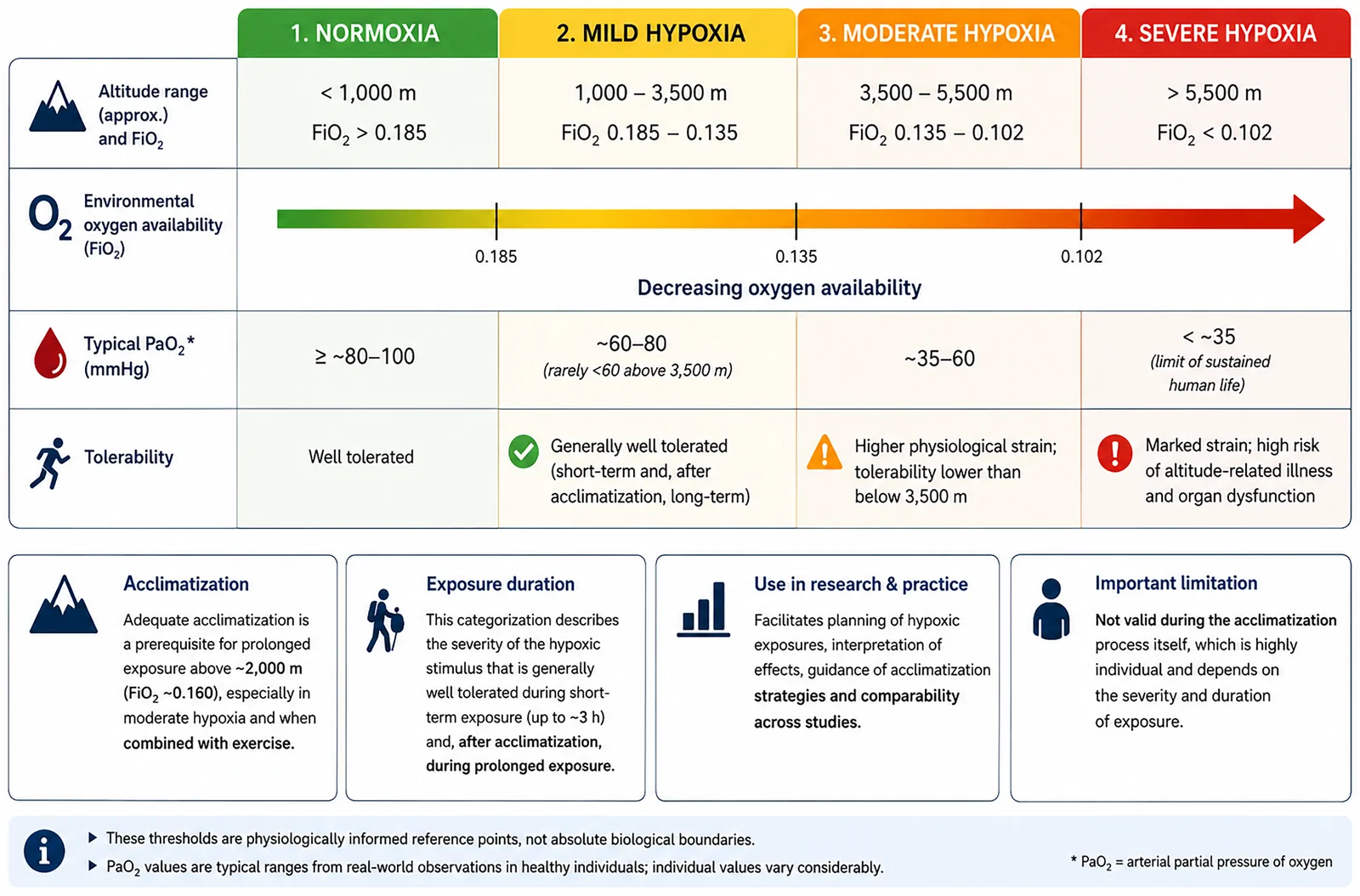
Proposed categorization of environmental hypoxia levels and suggested practical guidance.

Importantly, adequate acclimatization should be regarded as a prerequisite for prolonged exposure to hypoxia above approximately 2,000 m (FiO_2_: 0.160), particularly at moderate levels of hypoxia and when exposure is combined with physical exercise.

This proposed classification of hypoxia levels may facilitate the planning of hypoxic exposures, assessment of safety, interpretation of observed effects, guidance of acclimatization strategies, and comparability across studies.

## Data Availability

Publicly available datasets were analyzed in this study. This data can be found here: All data used are derived from publications cited in the paper.
